# Carbon-Based Nanomaterials as Sustainable Noble-Metal-Free Electrocatalysts

**DOI:** 10.3389/fchem.2019.00759

**Published:** 2019-11-12

**Authors:** Yuying Meng, Xiaoqing Huang, Huaijun Lin, Peng Zhang, Qingsheng Gao, Wei Li

**Affiliations:** ^1^Institute of Advanced Wear & Corrosion Resistant and Functional Materials, Jinan University, Guangzhou, China; ^2^Department of Chemistry, Jinan University, Guangzhou, China

**Keywords:** carbon-based nanomaterials, fuel cell, water splitting, energy-related reactions, doping effect

## Abstract

Nowadays, due to the worldwide growth demand of energy, over consumption of fossil fuel as well as their accompanying increased negative environmental impacts, the development of renewable energy systems, such as fuel cells and water electrolyzers, is becoming one of the “holy grail” for researchers. However, their large-scale applications have been severely limited by precious and unsustainable noble-metal electrocatalysts. Hence, it is highly desirable to develop robust electrocatalysts composed exclusively of low-cost and earth-abundant elements, to reduce or replace expensive and scarce noble-metals. Carbon-based nanomaterials, including heteroatoms-doped carbons and carbon-encapsulated metal materials, have recently attracted great interests because they show remarkable electrocatalytic performance and long-term stability for energy-related reactions, such as oxygen reduction reaction (ORR), hydrogen and oxygen evolution reactions (OER), hydrazine oxidation reaction (HzOR), etc. This review summarizes the recent progress in heteroatoms-doped carbon and carbon-encapsulated metal materials, highlighting the promise as cost-efficient electrocatalysts. Finally, a prospective on the future development of these promising materials is offered.

## Introduction

**Figure 1:**
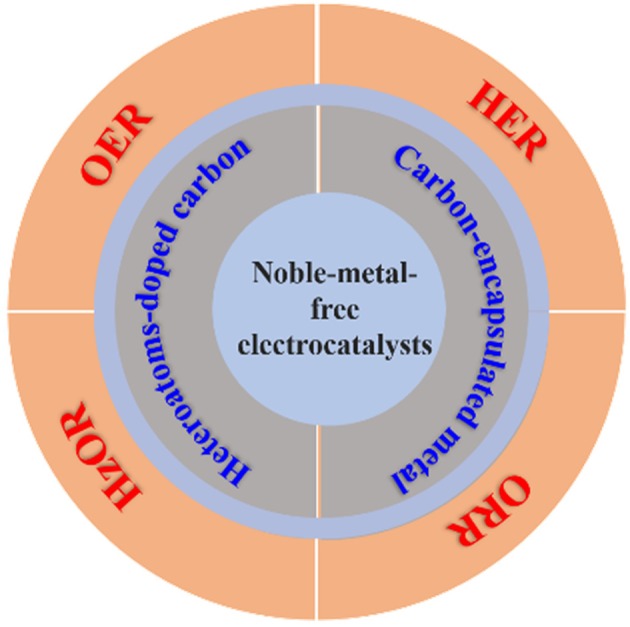
Summary illustration of the carbon-based noble-metal-free electrocatalysts and their applications.

The over consumption of fossil fuel reserves (natural gas, petroleum, coal, etc.) and their ongoing negative environmental impacts (e.g., water, air, and soil pollution) have driven the development of alternative, environmentally-friendly energy systems to reduce and/or eliminate our over-reliance on fossil fuels (Chow et al., 2003; Barnett et al., 2012; Subbaraman et al., 2012). Fuel cells, which can directly generate electricity from chemical fuels such as hydrogen and liquid hydrazine, and water electrolyzer that can generate the hydrogen fuel from water, are the promising energy conversion systems. However, the large-scale commercialization of fuel cells and water electrolyzer has so far been hampered by expensive and unsustainable electrocatalysts used for boosting the sluggish reactions at both anode and cathode, such as oxygen reduction reaction (ORR), hydrogen oxidation reaction (HOR), and water splitting including oxygen evolution reaction (OER) and hydrogen evolution reactions (HER) (Yang et al., 2008; Bhowmik et al., 2016; Cheng et al., 2016; Guo et al., 2016; Huang et al., 2018). In recent years, great efforts have been made to develop low-cost and earth-abundant electrocatalysts to promote the above-mentioned energy-related reactions, such as the carbon-based materials and transition metal sulfides and carbides (Feng et al., 2015; Hu et al., 2015; Liu et al., 2015; Huang C. et al., 2019; Wang X.-T. et al., 2019). Among the various electrocatalysts being investigated, carbon-based materials, including heteroatoms-doped carbons and carbon-encapsulated metal materials, have drawn increasing attentions due to their low-cost, high-efficiency, and good long-term durability (Figure 1). In this review, we will discuss the synthetic procedure of heteroatoms-doped carbons and carbon-encapsulated metal materials, as well as their characterizations and electrochemical performance.

## Fuel Cell Reactions

ORR is the reaction occurred on cathode in fuel cells, which involves multiple electrochemical processes. The ORR can either proceed through a two-step, a two electron (2e) process with the formation of ${\text{HO}}_{2}^{-}$ intermediate in alkaline, and H_2_O_2_ in acidic media, or via a more efficient four-electron (4e) pathway to directly reduce O_2_ into OH^−^ in alkaline and H_2_O in acidic media (Dai et al., 2015). The sluggish kinetic of ORR, the high cost and low durability of the Pt-based electrocatalysts are the current bottlenecks, which are needed to be addressed to enlarge the application of fuel cells (Liang et al., 2011; Huang et al., 2015b). Nitrogen and other heteroatoms-doped carbon materials, as well as carbon-encapsulated metal materials have shown remarkable electrocatalytic activity and durability toward ORR (Geng et al., 2011; Lin et al., 2012).

Hydrazine oxidation reaction (HzOR) is a vital reaction in direct liquid fuel cells (DLFCs) taking place at the anode. Due to its more favorable energy and power densities compared to hydrogen fuel, as well as no harmful and/or carbon-containing byproducts, HzOR has been becoming one of the great scientific and technological interest (Yamada et al., 2003; Sanabria-Chinchilla et al., 2011). Unfortunately, electrochemical conversion of hydrazine molecular to nitrogen on catalytic surface in DLFCs is kinetically sluggish and requires a relatively high over potential at electrodes, which is not well-suited for quantitation via conventional electrochemical approaches. In order to address the facing problem, extensively studies have been made in last decades to lower the overpotential of the hydrazine oxidation and improve the conversion efficiency. Currently, electrodes such as platinum, gold, silver, palladium, and alloys have been reported to be the electrocatalysts for the electrochemical oxidation of hydrazine (Gao et al., 2007; Rosca and Koper, 2008; Ye et al., 2008; Yi and Yu, 2009). However, these materials are tending to agglomerate or leach out during reaction and lose/reduce their activity. Others have found that carbon-based materials such as modified carbon nanotube and reduced graphene nanosheets can effectively electrocatalyze hydrazine oxidation, which paved a way to find the novel high-performance, inexpensive, sustainable metal-free HzOR electrocatalysts (Zhao et al., 2002; Wang et al., 2010).

## Water-Splitting Reactions

Electrolysis of water is widely considered to be the green and efficient approach to produce hydrogen fuel. The electrochemical water-splitting consists of two half reactions: the cathodic hydrogen evolution reaction (HER, 2H^+^ (aq) + 2e^−^ → H_2_ (g)) and the anodic oxygen evolution reaction (OER, 2H_2_O (l) → 4e^−^ + 4H^+^ (aq) + O_2_ (g)) (Shi and Zhang, 2016). To successfully conduct electrochemical water splitting, the applied voltages for both reactions must be above the thermodynamic potential values corresponding to the intrinsic activation barriers (i.e., overpotential, η) (Yan et al., 2014; Zou et al., 2014). Moreover, OER requires a higher overpotential (higher energy) to overcome the kinetic barrier to occur than that of HER due to the four-electron transfer involved in OER and gives the inherent sluggish kinetic (Xu et al., 2016). Hence, electrocatalysts for both the HER and OER are essential to reduce the overpotentials and consequently increase the energy conversion efficiency. Currently, the most effective electrocatalysts for the HER and OER are noble metal/metal oxides, such as Pt, RuO_2_, and IrO_2_ (Lee et al., 2012). However, the high cost and element scarcity of noble-metal based materials limited their wide application. Therefore, it is highly desired to develop effective alternative water splitting electrocatalysts with low cost and high abundance.

## Heteroatoms-Doped Carbons

As one of the most important classes of noble metal-free materials, carbon-based nanomaterials have drawn much attention because of their unique chemical, optical, electrical, and physical properties. Heteroatoms doping (e.g., nitrogen, boron, oxygen, sulfur, phosphorous) in carbon structure can affect various physicochemical properties of *sp*^2^ carbon materials, and hence lead to significant changes in local graphitic structure, hardness, electrical conductivity, and chemical reactivity (Wiggins-Camacho and Stevenson, 2009). Thereby, the structural incorporation of foreign atoms in graphitized carbon has received increasing attention nowadays due to its enhanced physicochemical properties and electrocatalytic performance.

**Figure 2:**
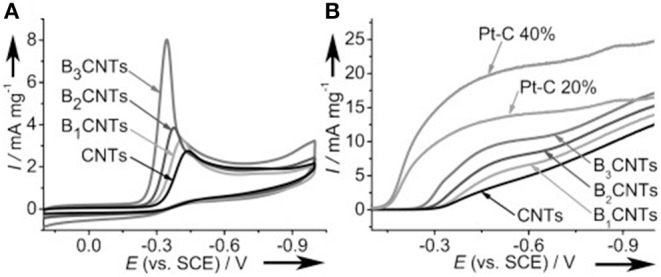
Electrocatalytic capabilities of the BCNT catalysts for the ORR in O_2_-saturated 1.0 M NaOH electrolyte. **(A)** CV curves (scan rate 50 mVs^−1^). **(B)** RDE voltammetry with a rotation speed of 2,500 rpm (scan rate 10 mV s^−1^). For comparison, corresponding examinations for CNTs and commercial Pt/C catalysts (20 and 40 wt % Pt loading) were also carried out. Re-printed with permission from Wiley-VCH Publications 2011 (Yang et al., 2011).

N-doped carbon materials, owing to their unique electronic properties and structural features, have been reported to exhibit not only efficient ORR performance comparable to that of commercial Pt/C (20 wt.%), but also good long-term stability, and excellent resistance to methanol crossover effects that are superior to Pt/C (Tang et al., 2009; Chen et al., 2012; Sharifi et al., 2012; Hou et al., 2015). The research area on this kind of metal-free electrocatalyst actually dates back to several decades ago, particularly to the seminal work reported by Dai, in which nitrogen doped carbon nanotube arrays were reported to remarkably electrocatalyze ORR in alkaline fuel cells with high long-term stability and good methanol crossover tolerance (Gong et al., 2009). Following this report, many other related materials without any metals, such as nitrogen-doped graphene and nitrogen-doped mesoporous carbons, were developed to show remarkable electrocatalytic activity and high durability for the ORR (Qu et al., 2010). Hu and his co-authors have prepared boron doped carbon nanotubes (BCNT) and directly used as electrocatalysts for ORR in alkaline medium (Yang et al., 2011). The result illustrated that the ORR performance of the synthesized BCN was related with the boron dopant amount. Specifically, the ORR activity increased with the increasing boron content, illustrating the importance of the boron moieties (Figure 2). Moreover, BCN gave good ORR selectivity and methanol crossover resistance, qualifying the B-doped nanotubes can serve as promising ORR metal-free electrocatalysts. Moreover, carbon materials with phosphorus dopants also have shown strikingly ORR activity in alkaline medium (Liu et al., 2011). Ordered mesoporous carbons with a small amount of P doping, prepared by Yu group, demonstrated their promise as a metal-free electrocatalyst for ORR, which featured excellent electrocatalytic activity via four-electron pathway in alkaline medium, enhanced stability, and excellent alcohol tolerance, compared to those of Pt/C (Yang D.-S. et al., 2012).

**Figure 3:**
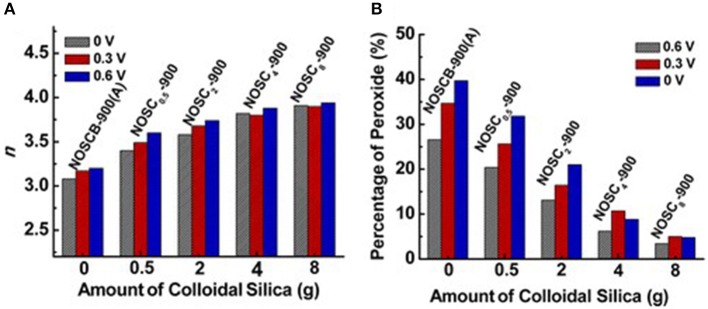
**(A)** Electron-transfer number (*n*) and **(B)** % peroxide formed at three different potentials vs. amount of colloidal silica used as templates for a series of NOSC_*x*_-900 materials. Re-printed with permission from the American Chemical Society Publications 2014 (Meng et al., 2014a).

Along with single heteroatoms doped carbon materials, dual or multi heteroatoms doped carbons, which constitute of two or three kinds of heteroatoms in the carbon, were also investigated as metal-free electrocatalysts for the ORR. Wang et al. reported that vertically aligned B and N co-doped carbon nanotubes electrode, which prepared by pyrolysis of melamine diborate, gave a higher electrocatalytic activity for ORR in alkaline media than the one singly doped with boron or nitrogen (Wang et al., 2011). They believed that the promoted activity was mainly attributed to the synergetic effect arising from the co-doping of boron and nitrogen. On the other hand, nitrogen and sulfur co-doped carbon aerogels have improved the overall electrocatalytic activity in both basic and acidic media, compared to the corresponding carbon solely doped with nitrogen, suggesting sulfur co-doping can further enhance the ORR activity (Wohlgemuth et al., 2012). Recently, researchers also reported the tri (N, B, P)-doped carbon can significantly improve the performance of nitrogen doped carbons and show remarkable ORR performance (Choi et al., 2012). We have synthesized N-, O-, and S-tridoped, polypyrrole-derived mesoporous carbons (NOSCs) and studied their ORR performance using colloidal silica as template. The synthesized NOSCs exhibited good catalytic activity toward ORR with low onset potential and low Tafel slope (Meng et al., 2014b). Moreover, the electron transfer numbers and H_2_O/H_2_O_2_ ratios as product of the electrocatalytic reaction were found to be tuneable by the amount of colloidal silica and their synergistic effect of N, S, and O tri-doping (Figure 3).

**Figure 4:**
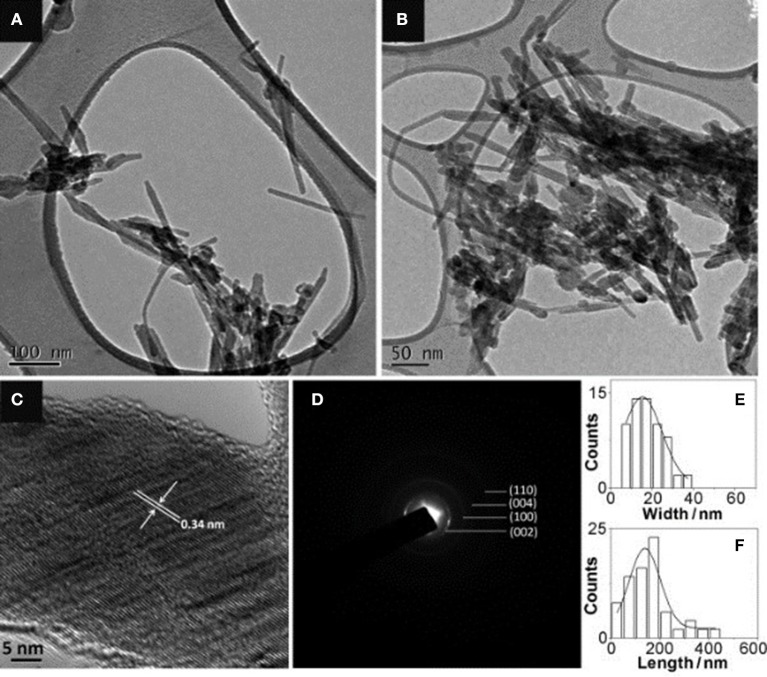
Characterization of N-doped carbon nanoneedles: **(A)** low magnification and **(B)** slightly higher magnification TEM images. **(C)** HRTEM and **(D)** SAED. **(E)** Their width distribution and **(F)** length distribution. Re-printed with permission from Wiley-VCH Publications 2012 (Silva et al., 2012).

**Figure 5:**
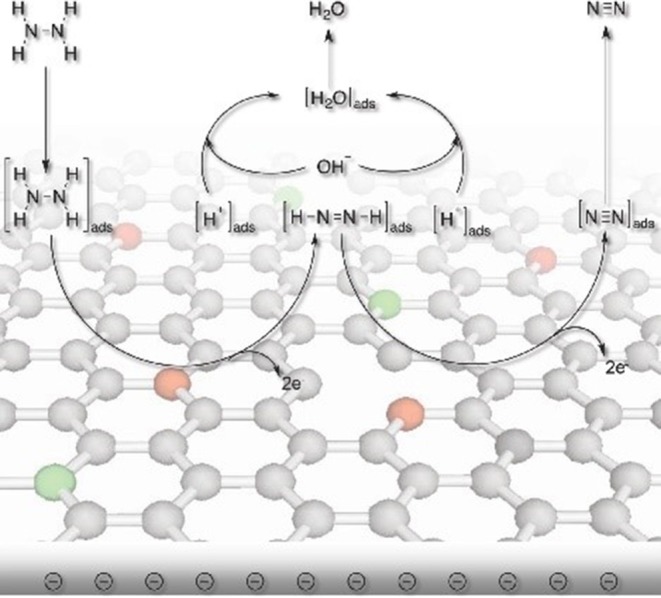
Schematic illustration showing the proposed electrocatalytic processes over nitrogen and oxygen doped carbon. Re-printed with permission from Wiley-VCH Publications 2014 (Meng et al., 2014b).

Additionally, heteroatoms doped carbons can also serve as metal-free electrocatalysts for HzOR, HER and OER. N-doped carbon nanoneedles (CNNs) with well-organized graphitic multi-layers and large proportions of N-doped edge planes (Figure 4) have been proven to be efficient metal-free electrocatalysts for hydrazine oxidation (Silva et al., 2012). The high catalytic activity toward HzOR was mostly due to their unique structures and large proportions of exposed edge planes. Later, the same group also synthesized other carbon-based materials by carbonization of cellulose filter paper, rice, yeast cell, and polypyrrole, all of which showed remarkable HzOR activity (Huang et al., 2015a; Koh et al., 2016; Martins et al., 2016). In the case of nitrogen and oxygen doped carbons, they believe that the presence of dopants and defects aid the effective adsorption of hydrazine onto the catalytic surfaces, then the hydrazine molecule was effectively dissociated/oxidized by the flow of electrons, giving a good electrocatalytic performance (Figure 5) (Meng et al., 2014b). During the oxidation process, carbon atoms with the relatively positive charge in the graphitic structure, facilitated the flow of electrons, which allowed the oxidation of N_2_H_4_ molecular to form N_2_ and H^+^. H^+^ was then react with OH^−^ to form water. Subsequently, the water and nitrogen products were desorbed and fresh hydrazine molecules was adsorbed on the surface to keep the catalytic cycle running.

With the high rapid development of graphene and carbon-based materials, heteroatoms doped carbons were also investigated as OER and HER electrocatalysts in water-splitting reactions. Zhao *et al* reported that the nitrogen-doped carbon materials function as the efficient oxygen evolution electrocatalysts with good stability (Zhao et al., 2013). The material gave a current density of 10 mA cm^−2^ at the overpotential of 0.38 V in alkaline electrolytes, which was comparable to those of iridium and cobalt oxide catalysts. The electrochemical and physical results displayed that the good oxygen evolution activity of the nitrogen/carbon materials was associated with the pyridinic-N and quaternary-N. Nitrogen and phosphorus co-doped mesoporous carbon foam with a large surface area of ~1,663 m^2^ g^−1^ was reported to act as bi-functional electrocatalyst for OER and ORR with good electrocatalytic properties (Zhang et al., 2015). The N and P co-doping and the highly porous network of the carbon foam were the main reason for the good bifunctional activity. Later, Dai et al. reported that N, S co-doped graphitic sheets with stereoscopic holes (SHG) can efficiently serve as tri-functional electrocatalysts for the ORR, OER, and HER, simultaneously (Hu and Dai, 2017). The SHG electrode showed a remarkable OER activity with a small onset potential of 1.49 V and overpotential of 1.56 V vs. RHE at a current density of 10 mA cm^−2^ in 0.1 M KOH electrolyte, which were comparable to those of commercially available RuO_2_ electrocatalyst, respectively. In the case of HER, SHG electrode exhibited a similar activity of metal-containing HER electrocatalysts, giving positive onset potential, large current density and small Tafel slope. The multifunctional electrocatalytic activities were attributed to the synergistic effect of N, S co-doping and the large surface area derived from this unique SHG architecture, which provided efficient pathways for electron and electrolyte/reactant transports. B-substitute graphene (B-SuG) was also found to electrocatalyze HER in acidic medium (Sathe et al., 2014). In comparison with the graphene without B doping, B-SuG performed a better activity, with more positive onset potential and larger current density. Jiao et al. have synthesized a series of heteroatoms doped graphene and investigated their electrochemical performance as metal-free electrocatalyst for HER (Jiao et al., 2016). Density functional theory (DFT) calculations illustrated that heteroatoms doping can significantly increase the hydrogen adsorption strength of graphene, lower the adsorption free energy of H (Δ${\text{G}}_{\text{H}}^{*}$) and thus enhance the HER activity, especially for the dual doping ones.

## Synthesis of Heteroatoms-Doped Carbon Materials

**Figure 6:**
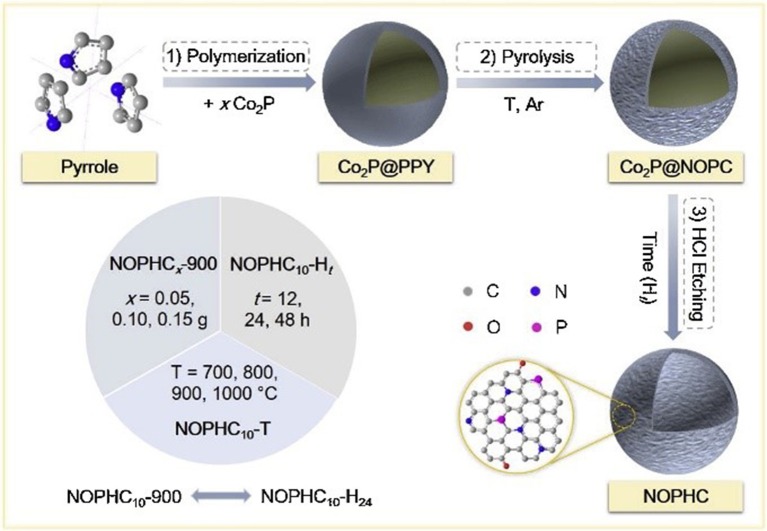
Synthetic procedure used to prepare NOPHCs electrocatalysts. Re-printed with permission from Elsevier Publications 2019 (Huang S. et al., 2019).

The preparation of heteroatom-doped carbon nanomaterials involves one-pot synthesis by *in-situ* incorporating heteroatoms during the formation of carbon nanomaterials or the post-synthesis through the post-treatment of performed carbon nanomaterials with the heteroatoms-containing precursor. The *in-situ* doping, direct carbonization of the dopant-containing precursors and carbon precursors together, can ensure the dopants structural incorporation into the carbon framework with a homogeneous distribution. In recent years, several heteroatom-doped carbon materials have been fabricated by this direct method. Nitrogen-doped carbon nanotube and nanofiber have been prepared from nitrogen-containing precursors, such as melamine (Terrones and Terrones, 1999; Terrones et al., 1999), benzylamine (Munoz-Sandoval et al., 2017), acetonitrile (Xia and Mokaya, 2004). Moreover, other synthetic substances, possessing large amount of dopants atoms, were also used to prepare heteroatoms-doped mesoporous carbons, including dicyandiamide (Liu R. et al., 2010; Liu Z.-W. et al., 2010), triphenylphosphine (Yang S. et al., 2012), polyaniline (Ajayan et al., 2007; Vinu et al., 2008; Lei et al., 2009), polypyrrole (Chang et al., 2007; Shrestha and Mustain, 2010), and so on. Asefa group has used polyaniline as the carbon and nitrogen precursor to synthesize the nitrogen and oxygen co-doped mesoporous carbons (PDMCs), which showed remarkable ORR performance with positive onset potential, large current density, high electron transfer number and long-term stability, due to the structural doping of heteroatoms into the carbon framework (Silva et al., 2013). Recently, our group have synthesized N, O, and P tri-doped hollow carbons (NOPHC), which served as bifunctional metal-free electrocatalysts for HER and ORR, using polypyrrole as precursor and Co_2_P as template (Huang S. et al., 2019). N, O and P were “*in-situ*” incorporated into the carbon structure during pyrolysis process, while hollow structure formed after removal of Co_2_P template (Figure 6). Additionally, Antonietti and others also employed non-volatile ionic liquids (ILs), composed of an organic N-containing cation and a bulky inorganic anion, as excellent direct precursors for synthesis of nitrogen (Paraknowitsch et al., 2010a,b), phosphorous (Paraknowitsch et al., 2013), and/or sulfur-doped (Wang and Dai, 2010; Paraknowitsch et al., 2012) carbon materials with high amount of dopants. Post-synthesis method was also widely employed to fabricate heteroatoms doped carbons. Yang and co-workers have successfully synthesized N (or S) doped graphene with large surface area via pyrolysis of graphene oxide in presence of guest gases (NH_3_ or H_2_S). When the annealing temperature was in the range of 500–1,000°C, both N and S were doped into the graphitic carbon, forming different binding configurations at the edges or on the planes of the graphene (Yang S. et al., 2012).

## Carbon-Encapsulated Metal Materials

**Figure 7:**
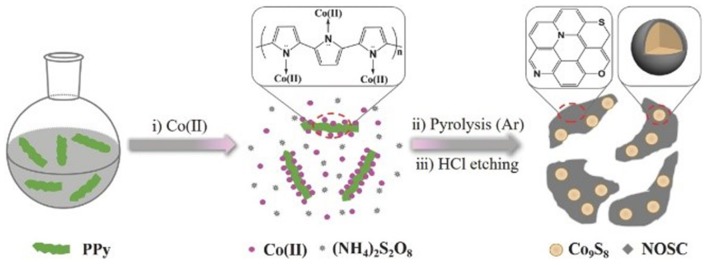
Synthetic procedures used to synthesize Co_9_S_8_@NOSC hybrid electrocatalysts. Re-printed with permission from Wiley-VCH Publications 2017 (Huang et al., 2017).

In addition to heteroatoms-doped carbons, carbon-encapsulated metals materials were also widely investigated as promising alternatives of the noble-metal based electrocatalysts for the energy conversion systems (Wang et al., 2018; Ouyang et al., 2019; Wang J.-Y. et al., 2019). Bao group has encapsulated 3d transition metals Fe, Co, and the FeCo alloy into nitrogen-doped carbon nanotubes (NCNTs) through a chemical vapor deposition (CVD) method (Deng et al., 2014). The synthesized NCNTs encapsulating 3d TM Fe, Co, and FeCo alloy nanoparticles (NPs) displayed good activity and long-term durability toward HER in acidic medium. DFT calculations, combining with the experimental results, illustrated that the introduction of metal and nitrogen in the carbon can synergistically enhance the HER activity. Our group also prepared N-, O-, and S-tridoped carbon-encapsulated Co_9_S_8_ materials (Co_9_S_8_@NOSCs) which have proven to act as noble metal-free bifunctional electrocatalysts for HER and OER in alkaline media (Huang et al., 2017). The Co_9_S_8_@NOSC nanocomposite materials were fabricated by preparing a S- and Co(II)-containing polypyrrole solid precursors (S-Co-PPY), carbonizing the S-Co-PPY precursor at different high temperatures, and then removal of surface bound metallic species on the carbonized products in concentrated HCl solution, as illustrated in Figure 7. Regarding to OER, Co_9_S_8_@NOSC-900 nanomaterials, obtained at pyrolysis temperature of 900°C, gave an efficient electrocatalytic activity, with a small overpotential of 340 mV at current density of 10 mA cm^−2^, high anodic current density, low Tafel slope (68 mV dec^−1^) as well as high (nearly 100%) Faradic efficiency. The excellent electrocatalytic activity can be derived from the synergistic effects between the heteroatom-doped carbon layers and the Co_9_S_8_ cores in the materials. Later, N-, O-, and S-doped carbon-encapsulated Ni_3_S_2_ and NiS core-shell architectures were prepared using the similar method with S- and Ni(II)-containing polypyrrole solid precursors (S-Ni-PPY) (Cao et al., 2018). The materials can bifunctionally electrocatalyze HER and ORR in alkaline media with good activity and long-term stability.

## Conclusions, Future Outlook, and Perspectives

Over the last few years, great effort has been made to develop the carbon-based materials as (noble) metal-free electrocatalyst for the ORR, HzOR, HER, and OER for fuel cells and water electrolyzer. Although huge progress has been achieved, there is still more research needed to be given for carbon-based materials. For example, the electrocatalytic performance for most (noble) metal-free electrocatalysts is difficult to compete the noble-based counterparts, which require further improvement. Also, the electrocatalytic mechanism and the exact active sites are not well-known, both of which are needed to be explored. In addition, there is difficulty in obtaining heteroatoms-doped carbon with high density of dopants.

To overcome the above-mentioned shortcomings, new precursors or synthetic methods are demanded to fabricate the carbons with high density of heteroatoms or large number of active sites, which in turn can obtain the carbon materials with enhanced catalytic activity. We believed that by combining various experimental approaches, state-of-the-art characterizations and powerful computational calculations, the new, low-cost (noble) metal-free carbon electrocatalysts with high electrocatalytic activity and clear catalytic mechanism can be accomplished for clean energy systems.

## Author Contributions

All authors listed have made a substantial, direct and intellectual contribution to the work, and approved it for publication.

### Conflict of Interest

The authors declare that the research was conducted in the absence of any commercial or financial relationships that could be construed as a potential conflict of interest.
